# Sensitive Detection of C-Reactive Protein by One-Step Method Based on a Waveguide-Mode Sensor

**DOI:** 10.3390/s20113195

**Published:** 2020-06-04

**Authors:** Hiroki Ashiba, Chiaki Oyamada, Kazuya Hosokawa, Koji Ueno, Makoto Fujimaki

**Affiliations:** 1 Sensing System Research Center, National Institute of Advanced Industrial Science and Technology (AIST), Tsukuba Central 5, 1-1-1 Higashi, Tsukuba, Ibaraki 305-8565, Japan; m-fujimaki@aist.go.jp; 2 Research Institute, Fujimori Kogyo Co., Ltd., 1-10-1 Sachiura, Kanazawa-ku, Yokohama, Kanagawa 236-0003, Japan; chiaki-oyamada@zacros.co.jp (C.O.); kazuya-hosokawa@zacros.co.jp (K.H.); 3 C&I Co., Ltd., 2004-1 Tamatori, Tsukuba, Ibaraki 300-3255, Japan; ukoji.candi@gmail.com

**Keywords:** immunoassay, one-step method, optical sensor, evanescent light, waveguide-mode resonance, gold nanoparticle

## Abstract

One-step biosensing methods enable the quick and simplified detection of biological substances. In this study, we developed a sensitive one-step method on the basis of a waveguide-mode sensor, which is an optical sensor utilizing waveguide-mode resonance and evanescent light. Streptavidin-conjugated and gold-nanoparticle-conjugated antibodies were reacted with a target substance and applied onto a biotinylated sensing plate. The target substance was detected by observing changes in sensor signals caused by binding the immunocomplex to the sensing surface. Performance of the developed one-step method was examined using a C-reactive protein (CRP) as a target substance. A sensor signal corresponding to the concentration of CRP was obtained. The minimal detectable CRP concentration of the developed method was 10 pM. The developed method greatly simplifies quantitative protein detection without reducing sensitivity.

## 1. Introduction

Biosensors are widely studied for applications in healthcare, medicine, and environmental measurements. Various sensitive methods, such as enzyme-linked immunosorbent assay (ELISA) and chemiluminescent enzyme immunoassay (CLEIA), were developed for detecting trace biological substances [1,2,3,4,5,6]. These methods employ various techniques to enhance sensitivity: for example, improving selectivity by the sandwich binding of antibodies, and enhancing signals using coloring or luminescence caused by enzyme reactions. Recently, a digital ELISA that could achieve single-molecule detection was developed [7,8,9]. Digital ELISA divides an analyte solution into many microfractions and dramatically improves the efficiency of signal enhancement by enzyme reaction. However, such sensitive methods include multiplexed step-by-step reactions and washing processes, and are thus cumbersome and time-consuming. A sensitive and quantitative one-step method can make the detection of biological substances in medicine or environmental measurement more useful and effective.

Various one-step methods were developed on the basis of various detection techniques [10,11,12,13,14,15]. For practical use, the method should satisfy requirements of high sensitivity, short measurement time, high stability of sensor chips, and compactness of instruments. In this study, a sensitive one-step method of quantitative protein detection was developed on the basis of an optical sensor that utilizes evanescent light. Among optical sensors using evanescent light [16,17,18,19,20,21], a waveguide-mode sensor [21,22,23,24] was employed because it had the above-mentioned features. The waveguide-mode sensor utilized waveguide-mode resonance excited in a slab waveguide at the surface of the sensing plate. Changes in complex refractive indices in the vicinity of the surface were detected with high sensitivity using waveguide-mode resonance. Concretely, changes in refractive indices and extinction coefficients (i.e., optical absorption) were detected by observing changes in resonance wavelength and reflectance, respectively, and were thus evaluated independently. The sensor was particularly sensitive to changes in extinction coefficient, and high sensitivity was obtained in the detection of colored substances or those using colored labels [22,23]. By using a streptavidin-conjugated antibody as a capture probe, a gold nanoparticle (AuNP)-conjugated antibody as a signal probe, and a biotinylated waveguide-mode sensing plate as a sensing surface, a detection method containing only a single mixing and reaction was established. C-reactive protein (CRP) [25] is a blood biomarker that indicates inflammation caused by infection or tissue injury, for which various sensors [11,26,27,28,29,30] and commercial ELISA kits [31,32,33,34,35] were developed. The performance of the developed method was examined using CRP as a target substance.

## 2. Detection Scheme

**Figure 1:**
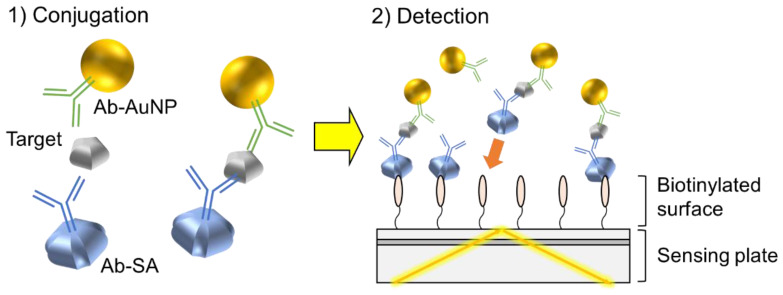
Detection scheme of one-step method. Ab-AuNP: gold nanoparticle-conjugated antibody; Ab-SA: streptavidin-conjugated antibody.

The detection scheme employed in this study is shown in Figure 1. For sensitive one-step detection, we designed a detection system that used streptavidin-conjugated antibodies as a capture probe, and AuNP-conjugated antibodies as a signal probe, where the AuNP was used as a colored label. Target substances, the capture probes, and the signal probes were mixed to form an immunocomplex, and the mixture was applied onto a biotinylated sensing plate for optical measurement using evanescent light. During these measurements, only substances placed in the vicinity of the surface of the sensing plate (i.e., several hundred nanometers from the surface) affected the magnitude of the signal. When immunocomplexes were captured at the surface of the sensing plate by biotin–streptavidin binding, reflectance at resonance wavelength was decreased due to the increased number of signal probes placed in the vicinity of the surface. The magnitude of the reflectance change was positively correlated with the number of bound immunocomplexes, which corresponded to the number of target substances. If there are no target substances, immunocomplexes cannot be formed, and only the capture probes bind to the surface. Since the binding of capture probes causes changes in refractive indices, but no changes in extinction coefficients, no changes in reflectance values were observed. Therefore, the target substances could be quantitatively detected by observing changes in the reflectance value.

For demonstration purposes, a CRP was used as the target substance. For the capture and signal probes, anti-CRP monoclonal antibody clone 6405 conjugated with streptavidin and anti-CRP monoclonal antibody clone 6404 conjugated with 20 nm of AuNP were used, respectively. As the diameter of AuNP increased, changes in reflectance generated by the single particle also increased. However, increases in background signal were measured due to AuNP gravitational sedimentation. We compared the background signal of AuNPs with diameters of 100, 60, and 20 nm. A diameter of 20 nm was chosen because the background signal was reduced to a negligible level (data are shown in Figure S1 in the Supplementary Materials). Conjugation of streptavidin and AuNP to the antibodies was carried out following the protocols of the labeling kits.

## 3. Materials and Methods

### 3.1. Materials

Phosphate-buffered saline (PBS) was purchased from Wako Pure Chemical Industries (Osaka, Japan). Tween 20 was purchased from Tokyo Chemical Industry (Tokyo, Japan); 3-aminopropyltriethoxysilane (APTES; LS-3150) was purchased from Shin-Etsu Chemical Co., Ltd. (Tokyo, Japan). Biotin-(AC_5_)_2_ Sulfo-Osu was purchased from Dojindo Laboratories (Kamimashiki, Japan). Recombinant human CRP (30-AC07) was purchased from Fitzgerald Industries International (North Acton, MA, USA). Monoclonal mouse antihuman CRP antibody clones 6404 and 6405 (Anti-h CRP clone 6404 SP-6 and 6405 SPTN-5) were purchased from Medix Biochemica (Espoo, Finland). The streptavidin-labeling kit (FastLink Streptavidin Labeling Kit) was purchased from Abnova Corporation (Taipei, Taiwan). The AuNP-labeling kit (20 nm NHS-Activated Gold Nanoparticle Conjugation Kit) was purchased from Cytodiagnostics Inc. (Burlington, Canada).

### 3.2. Apparatus

**Figure 2:**
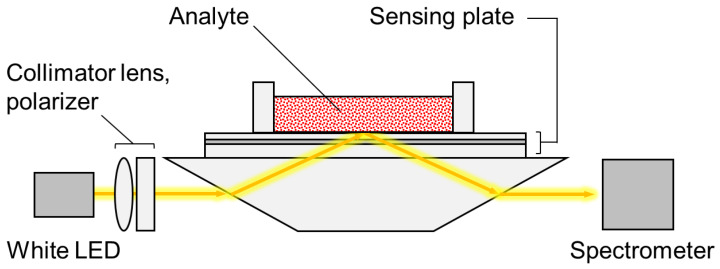
Schematic diagram of waveguide-mode sensor.

A four-channel waveguide-mode sensor Eva-M01 (C&I Co., Ltd., Tsukuba, Japan) was used as the sensing apparatus, which measured 15 × 6.5 × 6.5 cm in external dimensions. A schematic diagram of the optical system of the sensor is shown in Figure 2. The system was a parallel incident type optics based on the Kretchmann configuration [24]. Although only a single optical channel is depicted in Figure 2, four channels were placed in a direction perpendicular to the paper. Collimated, s-polarized white light was used to irradiate the surface of the sensing plate through a trapezoid prism. Total internal reflection of the white light occurred at the surface of the sensing plate, which resulted in the excitation of waveguide-mode resonance at a specific wavelength. Reflectance spectra were obtained with a spectrometer, and adsorption of target substances onto the sensing plate was detected by observing changes in the resonance conditions, including wavelength shifts and reflectance changes. In this study, changes in reflectance at the resonance wavelength (ΔR) were used to detect changes in extinction coefficients caused by the adsorption of AuNPs.

### 3.3. Sensor-Chip Preparation

Waveguide-mode sensor chip M01-35-40-325-01 (C&I Co., Ltd.) was used as the sensing plate. The waveguide, made of SiO_2_ and Si layers, was located at the surface of a glass substrate attached to the trapezoid prism with a bottom angle of 35 degrees, which was designed to excite waveguide-mode resonance at a wavelength of 530 nm. In order to prepare the biotinylated surface for target detection, the sensor chip was washed with ultrasonic cleaning using acetone and ethanol in sequence. The sensor chip was immersed in APTES solution 0.1 vol % in ethanol, and incubated for 24 h at room temperature. Subsequently, it was rinsed with acetone, and then cleaned ultrasonically using ethanol, NaOH 1 mM in H_2_O, HCl 1 mM in H_2_O, and ultrapure water in sequence. Then, the sensor chip was immersed in Biotin-(AC_5_)_2_ Sulfo-Osu 350 μg/mL solution in PBS and incubated for 24 h at room temperature. The sensor chip was rinsed with H_2_O containing 0.05 wt % Tween 20 and ultrapure water. A plastic frame with four through-holes was finally attached on the sensor chip to form four separate measurement chambers.

### 3.4. CRP Detection Protocol

Reagents, including CRP, and the capture and signal probes, were diluted with PBS containing 0.01 wt % Tween 20. CRP solutions, and the capture and signal probes were mixed and incubated for 30 min at room temperature when using the one-step method. Forty microliters of the incubated mixture were applied to the sensor chip, and ΔR was observed with the waveguide-mode sensor. Final concentrations of the capture and signal probes in the mixture were 2 nM and 200 pM, respectively.

For comparison purposes, another detection experiment was conducted using a step-by-step method. First, 2 nM of the capture probe solution was applied to the sensor chip and incubated for 5 min at room temperature. The surface of the sensor chip was rinsed with PBS containing 0.01 wt % Tween 20 after incubation, and the unbound capture probes were removed. Next, the CRP solution was applied to the sensor chip and incubated for 10 min at room temperature. Finally, the signal-probe solution was added to the sensor chip to reach a final concentration of 200 pM, and ΔR was observed with the waveguide-mode sensor.

For both detection methods, the four measurement chambers on the sensor chip were used for simultaneous measurements of four samples, including a blank solution. The values of ΔR obtained with the CPR solutions were compared to those obtained with blank solutions for each of the sensing plates. 

## 4. Results

**Figure 3:**
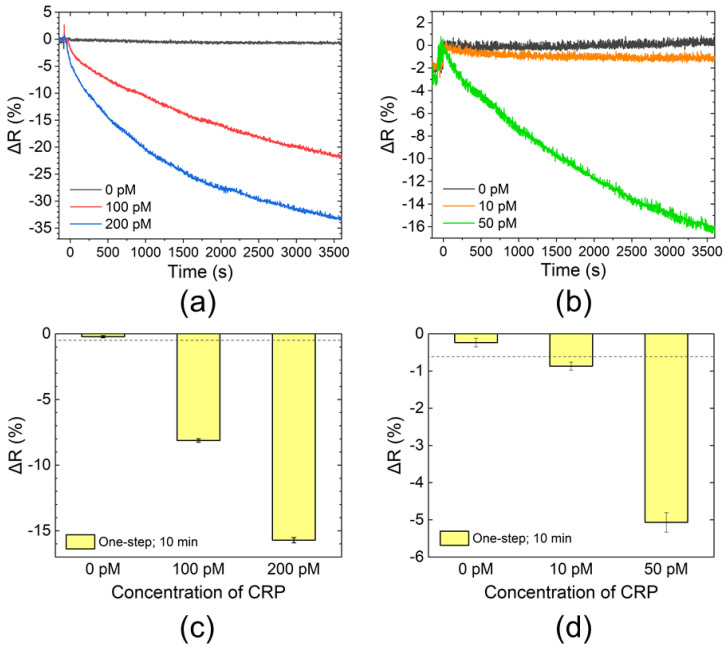
C-reactive protein (CRP) detection results using one-step method. Time course of changes in reflectance (ΔR) measured using waveguide-mode sensor for (**a**) higher and (**b**) lower concentration samples. Values of ΔR at 10 min for (**c**) higher and (**d**) lower concentration samples. Bars show mean values of 10 data points around 10 min presented in (**a**,**b**), respectively. Error bars are standard deviations (σ). Dashed lines show values of mean minus 3.3 σ of 0 pM sample (blank solution).

The ΔR vs. time values obtained using the one-step method are shown in Figure 3. The three datasets shown in Figure 3a,b were obtained at the same time on the same sensor chips (a streptavidin–AuNP conjugate was measured as a “positive control” using the one remaining chamber on the sensor chip. The positive control simply checked the sensor chip to be functional; data are not presented in the figure). The data-acquisition interval was 1 s. For the blank solution (0 pM), ΔR was almost zero. In contrast, samples containing CRP had decreased reflectance values over time. Therefore, the detection of CRP using the one-step method was successfully demonstrated. To determine ΔR of the CRP samples to be positive or negative, values of ΔR at 5, 10, 30, and 50 min were compared. Ten data points around each measurement time were taken to calculate means and standard deviations (σ) of ΔR (i.e., σ represents ΔR variation mainly caused by noise from the photodetector). The mean values of CRP samples were compared with the values of mean minus 3.3σ of the blank solution (because the compared values of ΔR were measured simultaneously on the same sensor chip, variances caused by the difference between sensor chips and ambient conditions were negligible). The results of positive/negative determination were the same for ΔR at 10, 30, and 50 min; therefore, the measurement time of 10 min was enough for CRP detection. The mean values and σ of ΔR at 10 min are shown in Figure 3c,d. The absolute values of ΔR were positively correlated with CRP concentration, and the minimal detectable CRP concentration of this measurement was 10 pM.

**Figure 4:**
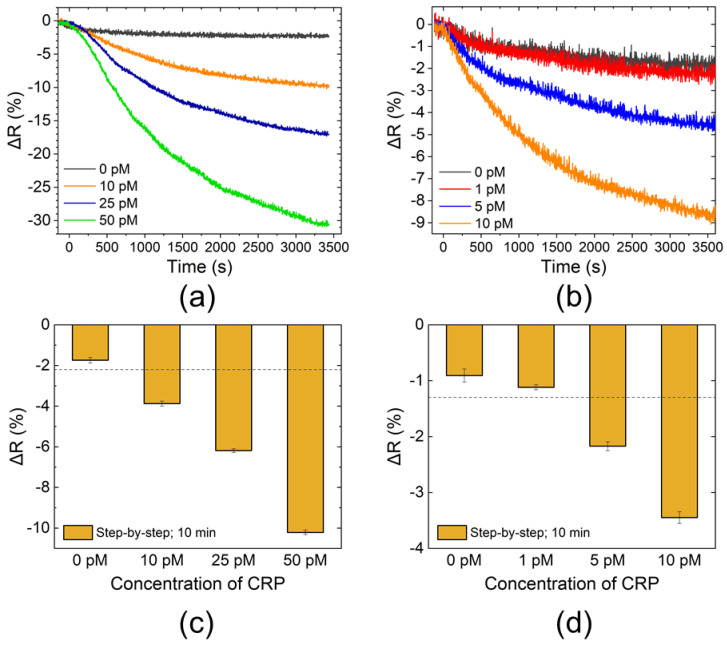
CRP detection results using step-by-step method. Time course of changes in reflectance (ΔR) measured using waveguide-mode sensor for (**a**) higher and (**b**) lower concentration samples. Values of ΔR at 10 min for (**c**) higher and (**d**) lower concentration samples. Bars show mean values of 10 data points around 10 min presented in (**a**,**b**), respectively. Error bars are standard deviations (σ). Dashed lines show values of mean minus 3.3 σ of 0 pM sample (blank solution).

The ΔR vs. time data obtained using the step-by-step method are shown in Figure 4. The four datasets shown in Figure 4a,b were obtained at the same time on the same sensor chips (the positive control was not included in these measurements). The mean values and σ of ΔR at 10 min, calculated in the same way as in the one-step method, are shown in Figure 4c,d. The minimal detectable concentration was 5 pM.

## 5. Discussion

CRP detection was successfully performed with both the one-step and the step-by-step methods. The step-by-step method exhibited slightly higher sensitivity than that of the one-step method. Although the concentrations of the capture and signal probes were identical, changes in reflectance of the step-by-step method were more extensive than those of the one-step method at the same CRP concentration (e.g., 10 and 50 pM in Figure 3 and Figure 4). Since CRP is a pentameric protein [25], several monoclonal antibodies of the same clone can bind to single CRP molecules. When CRP was reacted with the capture and signal probes in the one-step method, it was free in the solution; thus, undesired immunocomplexes of streptavidin–CRP–streptavidin or AuNP–CRP–AuNP could be formed along with the intended complex of streptavidin–CRP–AuNP. These undesired immunocomplexes would not contribute to the sensor signal. Therefore, changes in reflectance while using the one-step method would be smaller than those of the step-by-step method. However, the difference in sensitivity was minor, and the measurement process could be drastically simplified with the one-step method. In addition, if monomeric proteins were chosen as the target substance, basically only one monoclonal antibody of the same clone could bind to single protein molecules. Then, the formation of undesired immunocomplexes was avoided, and the sensitivity of the one-step method would be comparable to that of the step-by-step method. As for the sensitivity, the one-step method could be affected by free biotin molecules if present in samples, which bind the streptavidin of the capture probes and could inhibit the binding of the immunocomplex to the sensing surface. Blood, for example, contains intrinsic biotin, and direct measurements of blood samples using the one-step method would thus result in decreased sensitivity. Removal of biotin using, for example, streptavidin columns, is an effective preprocess when measuring biotin-containing samples.

In this study, the one-step method was developed on the basis of a waveguide-mode sensor. Similar methods could be developed by utilizing other optical sensors and evanescent light, such as surface plasmon resonance (SPR) sensors, on which AuNPs could be used as a label [36,37]. For comparing the waveguide-mode-based and SPR-based methods, a simulation based on the Fresnel equations was conducted using the transfer-matrix method [38]. Sensing plates of the waveguide-mode and SPR sensors were expressed by multilayer models for calculating reflectance spectra. The model of the waveguide-mode sensor was designed to represent experiment conditions: from bottom to top, an SiO_2_ substrate, an Si layer with a thickness of 40 nm, an SiO_2_ layer with a thickness of 325 nm, and water. An s-polarized light was used at an incident angle of 69.1°, which corresponded to the angle made by the prism employed in the experiment. Attachment of an Au layer at the surface of the SiO_2_ layer was tested, substituting the binding of AuNPs, to evaluate changes in spectra and ΔR. Values of refractive indices of SiO_2_ [39], Si [40], water [41], and Au [42] were taken from the literature. 

**Figure 5:**
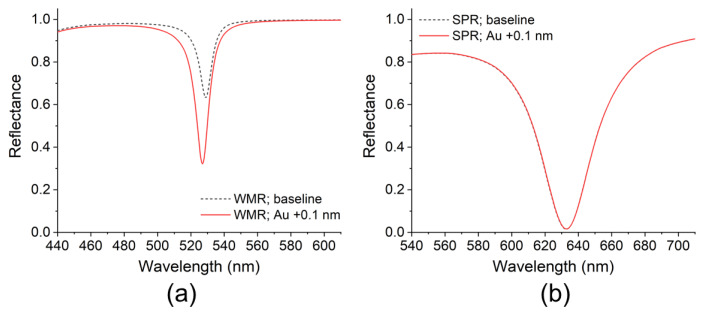
Simulation results of (**a**) waveguide-mode and (**b**) SPR sensors. Black broken and red solid lines represent before and after attachment of Au layer at surface of sensing plates, respectively.

The simulation results of the waveguide-mode sensor are shown in Figure 5a. A large change in the resonance dip in the spectra of ΔR = −31.0% resulted from the attachment of an Au layer with a thickness of 0.1 nm. For the SPR sensor, a model was designed to represent a common experiment setup: from bottom to top, a silica substrate, Au layer with a thickness of 50 nm, and water. P-polarized light was used at an incident angle of 82.0°, which resulted in SPR excitation at a wavelength of 633 nm. Similar to the waveguide-mode sensor, attachment of an Au layer at the surface was tested. The simulation results of the SPR sensor are shown in Figure 5b. The change in spectra caused by the attachment of an Au layer was minute. This result indicated that the waveguide-mode sensor was much more sensitive to the attachment of Au than the SPR sensor. Of course, the attachment of an Au layer differs from the binding of AuNPs in several ways, e.g., uniform or disperse presence at the surface, and different optical properties due to excitation of localized SPR on AuNPs. However, as a general tendency, the sensitivity of the waveguide-mode sensor to colored materials (i.e., materials with extinction coefficients) was higher than that of SPR sensor as shown in Figure 5 (other simulation results of the attachment of Au on SPR sensors that use Ag or Al thin films for exciting SPR are shown in Figure S2 in the Supplementary Materials. Results were similar to those shown in Figure 5b). Therefore, the waveguide-mode sensor was more advantageous than the SPR sensor for the one-step method using AuNPs.

The minimal detectable CRP concentration of the developed one-step method (10 pM = 1 ng/mL) was comparable to that of many commercial ELISA kits [31,32,33,34,35]. In contrast to ELISA, which requires multiplexed reaction and washing processes, the developed method could drastically simplify measurements without reducing sensitivity. The cost of consumption of reagents was similar between the developed method and ELISA. Moreover, in contrast to most of the conventional methods that use antibody-immobilized sensing surfaces, the one-step method employed a biotinylated surface. This means that the sensing surface of the one-step method can be universally used, whereas that of conventional methods is specific to the target substances. Therefore, the one-step method enables the effective manufacturing of sensing plates. Although the capture and signal probes of the one-step method are specific to target substances, manufacturing and storage of the probes can be much more effective than that of the antibody-immobilized sensing plates. The total assay time of the one-step method was 40 min, including 30 min of reaction time and 10 min of waveguide-mode measurement. Assay time was shorter than that of ELISA, and could be further shortened by optimizing reaction conditions. The sensor chips for the developed method were highly stable due to the chemical and mechanical properties of the waveguide-mode sensing plates made of SiO_2_ and Si, and the instrument is useable in various situations owing to its compactness (15 × 6.5 × 6.5 cm). The developed one-step method is of value as a quick, sensitive, and quantitative method of detection of biological substances.

## 6. Conclusions

A one-step method for sensitive biosensing was developed on the basis of a waveguide-mode sensor. By binding the target substance, the capture probe of the streptavidin-conjugated antibody, and the signal probe of 20 nm of the AuNP-conjugated antibody, the target was captured onto the sensing surface by biotin–streptavidin binding, and detected as changes in reflectance measured using the waveguide-mode sensor. The minimal detectable concentration of CRP obtained using the developed method was 10 pM. The assay time of this method could be further reduced by optimizing reaction conditions. Moreover, sensitivity can be further enhanced by the development of more effective signal probes in the future.

## Supplementary Materials

The following are available online at https://www.mdpi.com/1424-8220/20/11/3195/s1. Figure S1: evaluation results of the relationship between diameters of AuNPs and intensities of nonspecific signals; Figure S2: simulation results of attachment of an Au layer on SPR sensors using Ag and Al thin films.
