# Supplementary material for: Sensitive Detection of C-Reactive Protein by One-Step Method Based on a Waveguide-Mode Sensor
Source: Sensors (Basel). 2020 Jun 4;20(11):3195. doi: 10.3390/s20113195 (PMC7309159; doi:10.3390/s20113195)
Supplement: Supplementary file 1 [file sensors-20-03195-s001.zip › Supplementary/sensors-797518-Supplementary.pdf]

sensors

MDPI

Letter

Sensitive Detection of C-Reactive Protein by One-Step Method Based on a Waveguide-Mode Sensor

Hiroki Ashiba [1,*], Chiaki Oyamada [2], Kazuya Hosokawa [2], Koji Ueno [3] and Makoto Fujimaki [1]

1 Sensing System Research Center, National Institute of Advanced Industrial Science and Technology (AIST), Tsukuba Central 5, 1-1-1 Higashi, Tsukuba, Ibaraki 305-8565, Japan; m-fujimaki@aist.go.jp
2 Research Institute, Fujimori Kogyo Co., Ltd., 1-10-1 Sachiura, Kanazawa-ku, Yokohama, Kanagawa 236-0003, Japan; chiaki-oyamada@zacros.co.jp (C.O.); kazuya-hosokawa@zacros.co.jp (K.H.)
3 C&I Co., Ltd., 2004-1 Tamatori, Tsukuba, Ibaraki 300-3255, Japan; ukoji.candi@gmail.com
* Correspondence: h.ashiba@aist.go.jp; Tel.: +81-29-861-4739

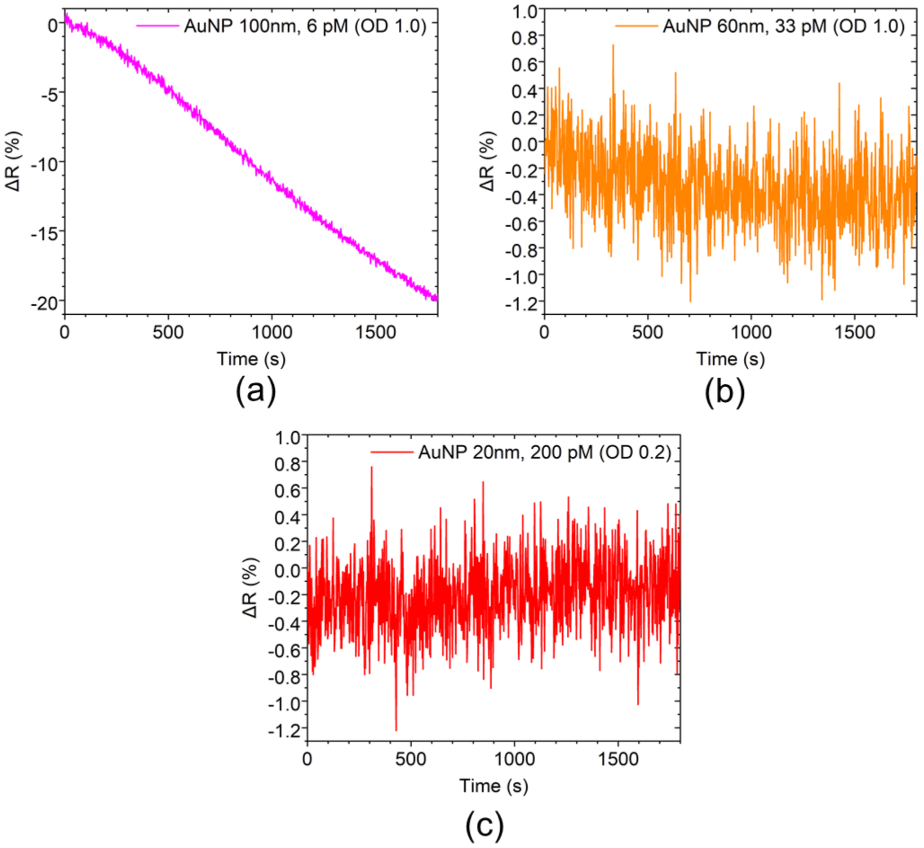

Figure S1. Evaluation results of relationship between diameters of AuNPs and intensities of background signals. Diameters and concentrations of tested AuNPs were 100 nm and 6 pM (OD 1.0) (**a**), 60 nm and 33 pM (OD 1.0) (**b**), and 20 nm and 200 pM (OD 0.2) (**c**), respectively. A single-channel waveguide-mode sensor was used for this test. Sensing plates were coated with a blocking agent, methoxytriethyleneglycol triethoxysilane (M3EG) [S1], to avoid adsorption of AuNPs. Dispersions of AuNPs without antibody immobilization were placed on sensing plates. Changes in reflectance at resonance wavelength (ΔR) were observed with a data-acquisition interval of 2 s. AuNPs with a diameter of 100 nm exhibited a large background signal that was attributed to gravitational sedimentation. AuNPs with a diameter of 60 nm also exhibited a slight background signal. AuNPs with a diameter of 20 nm exhibited negligible background signal; thus, diameter of 20 nm was chosen in this study.

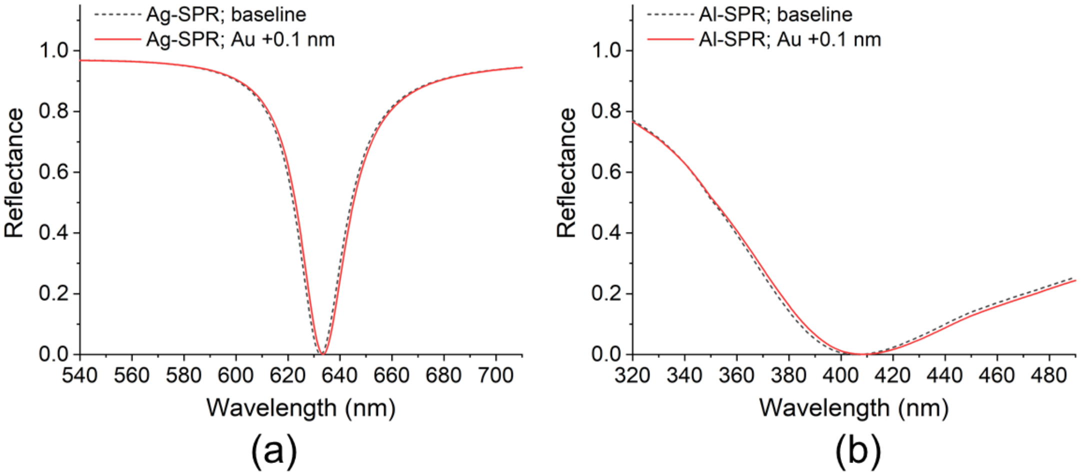

Figure S2. Simulation results of attachment of an Au layer on SPR sensors using Ag (**a**) and Al (**b**) thin films. Black broken and red solid lines represent before and after attachment of Au layer, respectively. The calculation method is the same as that of the simulation shown in the main text. The calculation model of Ag–SPR sensor was, from bottom to top, a silica substrate, Ag layer with a thickness of 57 nm, and water; p-polarized light was irradiated at incident angle of 74.1°, which resulted in SPR excitation at wavelength of 633 nm. Calculation model of Al–SPR sensor was, from bottom to top, a silica substrate, Al layer with a thickness of 19 nm, and water; p-polarized light was irradiated at an incident angle of 73.0°, which resulted in SPR excitation at wavelength of 405 nm. Values of refractive indices of Ag and Al were taken from [S2,S3], respectively. Changes in spectra of Ag– and Al–SPR sensors by attachment of an Au layer were minute compared with that of the waveguide-mode sensor (Figure 5a in main text), in the same way to the Au–SPR sensor (Figure 5b in main text).

References

S1. Tanaka, M.; Yoshioka, K.; Hirata, Y.; Fujimaki, M.; Kuwahara, M.; Niwa, O. Design and Fabrication of Biosensing Interface for Waveguide-Mode Sensor. *Langmuir* **2013**, 29, 13111–13120. https://doi.org/10.1021/la402802u.

S2. Babar, S.; Weaver, J.H. Optical constants of Cu, Ag, and Au revisited. Appl. Opt. 2015, 54, 477–481. https://doi.org/10.1364/AO.54.000477.

S3. Rakić, A.D. Algorithm for the determination of intrinsic optical constants of metal films: application to aluminum. *Appl. Opt.* **1995**, 34, 4755–4767. https://doi.org/10.1364/AO.34.004755.
